# Toll-Like Receptor 7 Agonist Therapy with Imidazoquinoline Enhances Cancer Cell Death and Increases Lymphocytic Infiltration and Proinflammatory Cytokine Production in Established Tumors of a Renal Cell Carcinoma Mouse Model

**DOI:** 10.1155/2012/103298

**Published:** 2012-02-22

**Authors:** Eric C. Kauffman, Huixian Liu, Michael J. Schwartz, Douglas S. Scherr

**Affiliations:** ^1^Department of Urology, Weill Medical College of Cornell University, New York, NY 10065, USA; ^2^Urologic Oncology Branch, National Cancer Institute, National Institutes of Health, Bethesda, MD 20892, USA; ^3^Smith Institute of Urology, North Shore-Long Island Jewish Health System, New Hyde Park, NY 11042, USA

## Abstract

Imidazoquinolines are synthetic toll-like receptor 7 and 8 agonists and potent dendritic cell activators with established anticancer activity. Here we test the hypothesis that imidazoquinoline has *in vivo* efficacy within established renal cell carcinoma (RCC) tumors. Immunocompetent mice bearing syngeneic RCC xenografts were treated with imidazoquinoline or placebo at two separate time points. Harvested tumors were assayed by TUNEL/caspase-3/Ki67 immunostains to evaluate cell death/apoptosis/proliferation, and CD3/B220/CD45 immunostains to evaluate T-cell lymphocyte/B-cell lymphocyte/pan-leukocyte tumor infiltration. ELISA measurement of tumor and serum levels of proinflammatory cytokines, IL-6 and MCP-1, was performed. A single imidazoquinoline dose significantly decreased RCC tumor growth by 50% and repeat dosing compounded the effect, without observed weight loss or other toxicity. Tumor immunostaining revealed significant increases in cell death and apoptosis without changes in cell proliferation, supporting induction of apoptosis as the primary mechanism of tumor growth suppression. Imidazoquinoline treatment also significantly enhanced peritumoral aggregation and intratumoral infiltration by T-cell lymphocytes, while increasing intratumoral (but not serum) levels of proinflammatory cytokines. In conclusion, imidazoquinoline treatment enhances T-cell lymphocyte infiltration and proinflammatory cytokine production within established mouse RCC tumors, while suppressing tumor growth via induction of cancer cell apoptosis. These findings support a therapeutic role for imidazoquinoline in RCC.

## 1. Introduction

Kidney cancer is responsible annually for over 58,000 new diagnoses and 13,000 deaths in the US [1]. Approximately 85% of these cancers are renal cell carcinomas (RCCs) arising from the renal tubule epithelial lining. RCC includes distinct histologic subtypes defined by different clinical behaviors, the most common being clear cell (65%), papillary (15%), and chromophobe RCC (5%). Despite earlier stage of detection in recent decades, RCC patient mortality has not decreased accordingly, and recurrence following definitive local therapy by surgery or ablative techniques remains a significant clinical challenge [2]. For patients presenting or recurring with RCC metastases, prognosis is poor, with a 5-year survival of less than 10% [3]. While multityrosine kinase inhibitors have shown recent promise with frequent clinical responses, complete responders are lacking, and there is question regarding survival benefits and durability of response [4, 5]. 

Interest in immunotherapy for RCC patients was ignited four decades back with the suggestion that immune cell activation mediates the occasional spontaneous regression of RCC pulmonary metastases [6]. The concept that the immune system may mediate RCC tumor suppression has gained more recent support from clinical success of immunotherapies in treating patients with metastatic RCC. To date, IFN-alpha and IL-2 remain among the most successful treatments for metastatic clear cell RCC, with modest survival benefits in prospective randomized trials and an approximately 5% incidence of durable complete response [7, 8]. The more recent demonstration of cancer-specific antigens recognized by T-cell lymphocytes found in RCC patient tumors has further supported the role of the immune system in this disease [9]. 

Relative to other solid tumor cancers, RCC tumors are highly immunogenic, as evidenced by abundant cytotoxic T-cell lymphocyte infiltrates showing specificity for autologous RCC cells [9, 10]. Despite their immunogenicity, the plasticity of RCC tumors enables them to escape immune destruction by what is increasingly believed to involve a variety of escape mechanisms [11]. Specific defects in T-cell lymphocytes from RCC patients have been identified and include dysregulated signaling pathways and increased apoptotic tendency [12]. Central to this immune breakdown may be the ability of some immunogenic tumors to block activation of dendritic cells, the primary antigen-presenting cells responsible for downstream effector T-cell lymphocyte activation [13]. Targeting dendritic cell activation may therefore provide an effective therapeutic strategy for clinical RCC.

Imidazoquinolines are synthetic immunomodulatory drugs that act by binding toll-like receptors 7 and 8 (TLR7/TLR8) on dendritic cells, structurally mimicking these receptors' natural ligand, viral single-stranded RNA [14, 15]. Whereas TLR8 is expressed in humans, only TLR7 is expressed in both humans and mice. Activation of TLR7 induces dendritic maturation through the MyD88/NF-kappaB signaling pathway, enhancing antigen presentation and downstream activation of antigen-specific T-cells, with profound elaboration of proinflammatory cytokines and chemokines including IFN-alpha, IL-6, IL-12, TNF-alpha, and MCP-1/CCL2 [15–19]. The net result is a coordinated immune response with potent antiviral and antitumor effects [13]. The best characterized imidazoquinoline to date, imiquimod, has demonstrated clinical efficacy against human papilloma virus/genital condyloma, basal cell carcinoma, and actinic keratosis and is approved by the Food and Drug Administration for topical treatment of these diseases. Additionally, there is a growing body of clinical evidence that imiquimod is effective in the treatment of other dermatologic malignancies, including melanoma and squamous cell carcinoma, with complete responses reported [20–22]. 

We have previously demonstrated a novel imidazoquinoline, 3M011, has *in vivo *efficacy against bladder tumorigenesis as well as systemically disseminated RCC cells in a metastatic mouse model [23, 24]. However, imidazoquinoline activity in established primary RCC tumors and its effects on lymphocyte infiltration or cytokine production in this cancer type have not yet been explored. Here, we employ a syngeneic xenograft mouse model to test the hypothesis that imidazoquinoline therapy has *in vivo* efficacy in established RCC primary tumors with regard to anticancer activity, lymphocytic infiltration, and intratumoral proinflammatory cytokine production. 

## 2. Materials and Methods

### 2.1. Cell Line

Experiments were performed using the kidney cancer cell line, RENCA, which was originally derived from a spontaneous RCC tumor in the BALB/c mouse strain. Cells were grown *in vitro* in RPMI media supplemented with L-glutamine (2 mM), penicillin (100 U/mL), streptomycin (100 U/mL), and 10% fetal bovine serum. Cell cultures were maintained at 37°C and 5% CO_2_. 

### 2.2. Mouse Model and Treatments

**Figure 1:**
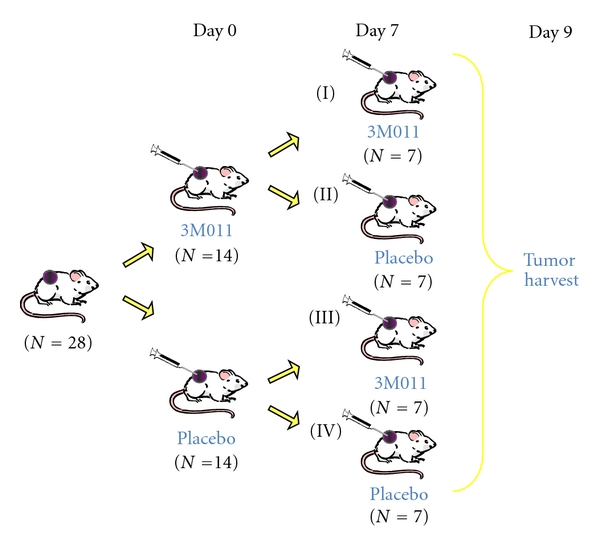
Generation of four mouse treatment groups. *N* = 28 mice harboring RENCA RCC tumor xenografts were randomized on day 0 and again on day 7 to treatment with either 3M011 imidazoquinoline or placebo. Four treatment groups (*N* = 7) were thus generated based on day 0/day 7 treatments: (I) 3M011/3M011, (II) 3M011/placebo, (III) placebo/3M011, and (IV) placebo/placebo.

Syngeneic RCC primary tumor xenografts were generated by injection of 2 × 10^5^ RENCA cells subcutaneously in the left flank of *N* = 28 eight-week-old female Balb/c mice (Jackson Laboratory, Bar Harbor, ME), giving rise to palpable tumors by two weeks after injection. Mice were randomized at 17 days after inoculation (defined as Day 0) to treatment by intratumoral injection with 0.1 mL of either 0.5 mg/mL (~2.5 mg/kg) 3M011 imidazoquinoline (3 M Pharmaceuticals, St. Paul, MN) or placebo control (sodium citrate vehicle, 0.03 M, pH 6.0). One week after initial treatment (Day 7), mice were rerandomized to receive either 3M011 or placebo injection. Four treatment arms were thus generated based on day 0/day 7 treatments (Figure 1). Mouse weights and tumor volumes were measured every 1-2 days, with volumes calculated as 0.4 × (width)^2^ × (length). Mice were sacrificed on day 9, and tumor weights were compared among treatment groups. Tumors and serum were harvested for immunohistochemistry and ELISA assays described below.

### 2.3. ELISA

Protein extract from harvested mouse tumors was obtained by mechanical homogenization of 100 mg tumor in 500 *μ*L of PBS supplemented with 1 mM protease inhibitor phenylmethylsulfonyl fluoride (PMSF) and a mild detergent (Triton X-100, 0.05%). Tumor protein extract and mouse serum were assayed by ELISA for levels of IL-6 and MCP-1 (Biolegend, San Deigo, CA). Briefly, 96-well plates were coated overnight with anti-mouse IL-6 or MCP-1 capture antibody at 4°C. Serum or tumor extract supernatant were added to wells after 1 : 5–1 : 20 dilution, followed by successive incubations at room temperature with anti-mouse IL-6 or MCP-1 detection antibody and horse radish peroxidase-conjugated anti-mouse secondary antibody. Color reactions were generated with the addition of peroxidase substrate and terminated after 15 minutes with 2 N H_2_SO_4_. Absorbance at 450 nm wavelength was measured, and protein concentration was determined by interpolation onto absorbance curves generated by recombinant IL-6 and MCP-1 protein standards.

### 2.4. Tumor Immunohistochemistry

 Following tissue removal for ELISA, the remaining harvested tumor tissue was fixed in 10% formalin solution, embedded in paraffin, and sectioned for immunohistochemical staining. Sections were heat deparaffinized at 58–60°C for 30 minutes. For TUNEL (Terminal deoxynucleotidyl transferase-mediated dUTP Nick End Labeling) staining identification of nonviable cells, antigen retrieval was performed by 15-minute incubation with 20 *μ*g/mL proteinase K (Sigma, St. Louis, MO) in 10 mM TRIS-HCl, pH 8.0, followed by endogenous peroxidase quenching with 3% hydrogen peroxide in PBS. TUNEL staining was then performed for one hour at 37°C with 2.0 units/*μ*L terminal transferase (Roche, Indianapolis, IN), 0.2 nmol biotin-16-dUTP (Roche, Indianapolis, IN), 2.5 mM cobalt chloride (Roche, Indianapolis, IN), 0.1 M sodium cacodylate pH 7.0 (Sigma, St. Louis, MO), 0.1 mM DTT (Sigma, St. Louis, MO), and 0.05 mg/mL bovine serum albumin (Sigma, St. Louis, MO). All other antigen retrieval was performed by microwaving at high power in 10 mM sodium citrate buffer (Sigma, St. Louis, MO), with the exception of B220 immunostaining, for which no antigen retrieval step was performed. After antigen retrieval, tumor sections were preincubated in 10% goat serum (MP Biomedicals) for 30 minutes at room temperature followed by primary antibody dilution in 2% bovine serum albumin overnight at 4°C. Specific primary antibodies and dilutions included goat anticleaved caspase 3 (Cell Signaling Cat. no. 9661) at 1 : 250 dilution (apoptotic cell identification); goat anti-Ki67 (Vector Laboratories Cat. no. VP-K451) at 1 : 10,000 dilution (cell proliferation identification); rabbit anti-CD3 (Dako Cat. no. A0452) at 1 : 1000 dilution (T-cell lymphocyte identification); rat anti-B220 (BD Pharmingen Cat. no. 550286) at 1 : 200 dilution (B-cell lymphocyte identification); rat anti-CD45 (BD Pharmingen Cat. no. 550539) at 1 : 10 dilution (pan-leukocyte identification). Sections were then incubated with secondary antibody for 30 minutes at room temperature, using biotinylated goat anti-rabbit IgG (Vector Labs BA-1000) at 1 : 1000 dilution for cleaved caspase-3, Ki67, and CD3, or biotinylated rabbit anti-rat IgG (Vector Labs BA-4001) at 1 : 100 dilution for CD45 and B220. For color stain reaction, sections were incubated with Vectastain Avidin-Biotin Complex Elite (Vector Labs) diluted 1 : 25 in PBS for 30 minutes, followed by 3,3-diaminobenzidine (Sigma, St. Louis, MO) and hematoxylin counterstain. Appropriate positive and negative control tissues (e.g., mouse spleen, mouse lymph nodes) were stained simultaneously. For all nonleukocytic markers, tumor stains were scored as the percentage of positively staining tissue. For leukocyte markers (CD3, CD45, and B220), the tumors were scored 0+ to 4+ separately for (1) peritumoral/capsular staining and (2) intratumoral staining. A maximum score (4+) for peritumoral (capsular) staining was given for leukocyte staining at least several cell layers thick and diffuse around the entire tumor edge. A maximum score (4+) for intratumoral staining was given for infiltrating leukocytes detectable within the tumor at close to a 1 : 1 ratio with surrounding cancer cells. 

### 2.5. Statistical Analysis

 Statistical comparisons of mean values were performed using a Student's T-test. Only *P* values < 0.05 were considered significant.

## 3. Results

### 3.1. Imidazoquinoline Effect on RCC Tumor Growth

**Figure 2:**
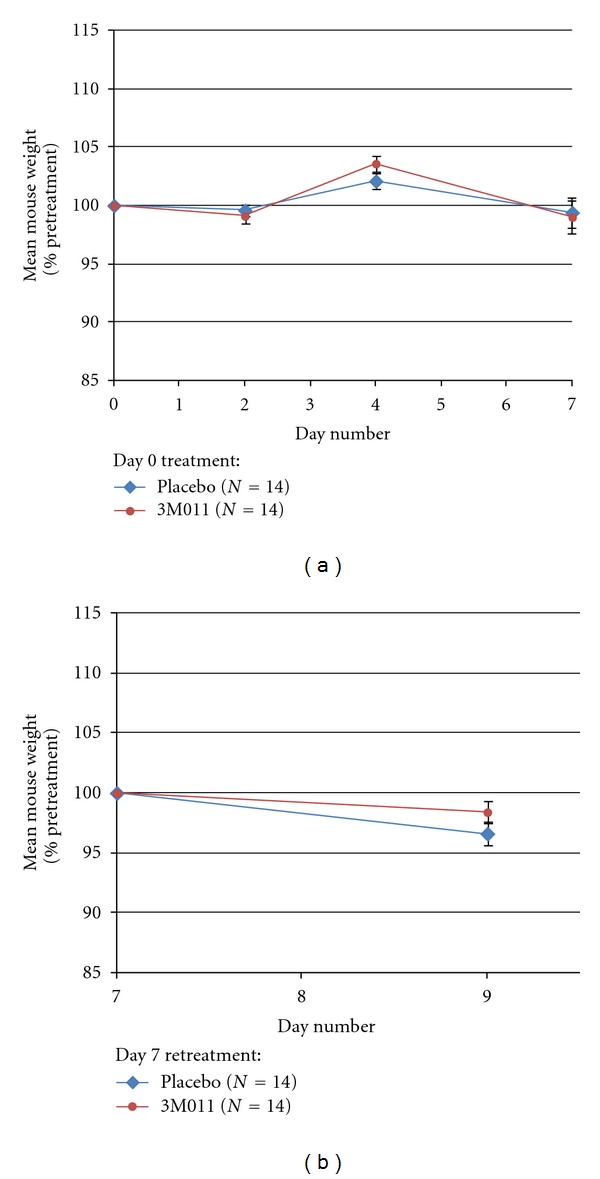
Intratumoral 3M011 injection does not alter mouse weight. (a) Mean percent change in mouse weight following 3M011 versus placebo treatment on day 0 (*P* = 0.47, 0.14, and 0.82 at 2, 4, and 7 days, resp.). (b) Mean percent change in mouse weight following 3M011 versus placebo treatment on day 7 (*P* = 0.13). Error bars = standard error of the mean.

 Mice harboring syngeneic RCC primary tumor xenografts (*N* = 28) were treated on day 0/day 7 with imidazoquinoline/imidazoquinoline (*N* = 7), imidazoquinoline/placebo (*N* = 7), placebo/imidazoquinoline (*N* = 7), or placebo/placebo (*N* = 7) (Figure 1). Imidazoquinoline treatment was well tolerated with no episodes of shivering, tremors, or raised fur. Mice remained active with weights well maintained following both initial and repeat dosing (Figures 2(a) and 2(b)).

**Figure 3:**
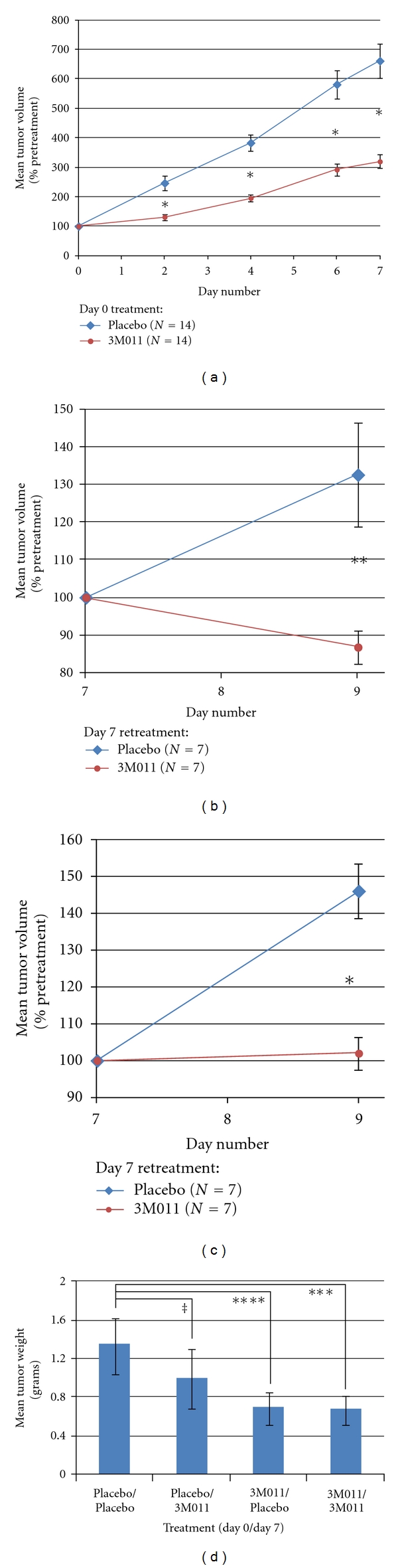
3M011 reduces RCC tumor growth in mice. RENCA RCC xenografts in mice (*N* = 28) were treated on day 0 and day 7 with 3M011 imidazoquinoline or placebo. (a) Tumor growth following 3M011 versus placebo treatment on day 0. (b) Tumor growth following retreatment with 3M011 versus placebo on day 7 in mice receiving placebo on day 0. (c) Tumor growth following retreatment with 3M011 versus placebo on day 7 in mice receiving 3M011 on day 0. (d) Mean weights of tumors harvested on day 9 based on day 0/day 7 treatment. *P < 0.001; **P < 0.01; ***P < 0.05; ****P < 0.10; ^‡^ *P* = 0.45; error bars = standard error of the mean.

 Mean tumor size at initial treatment was 0.13 cm^3^, with no significant difference between imidazoquinoline and placebo arms. As shown in Figure 3, a single dose of imidazoquinoline on day 0 significantly decreased RCC tumor growth rate over the following week relative to placebo, yielding a relative ~50% reduction in tumor volume at all subsequently measured time points (Figure 3(a)). Delaying the first dose of imidazoquinoline until day 7, when tumors were on average >600% larger, still resulted in significant inhibition of tumor growth, causing partial tumor regression over the following 48 hours despite a 32% increase in placebo-treated tumor size (*P* < 0.01) (Figure 3(b)). Mice receiving their second dose of imidazoquinoline on day 7 showed a continued response, with no tumor growth over the following 48 hours despite 46% enlargement of control tumors (*P* < 0.005) (Figure 3(c)). Weights of tumors harvested on day 9 were 50% lower in mice treated on day 0 with imidazoquinoline compared to those receiving placebo (*P* < 0.001) (Figure 3(d)). 

### 3.2. Imidazoquinoline Effect on RCC Tumor Cell Death and Proliferation

**Figure 4:**
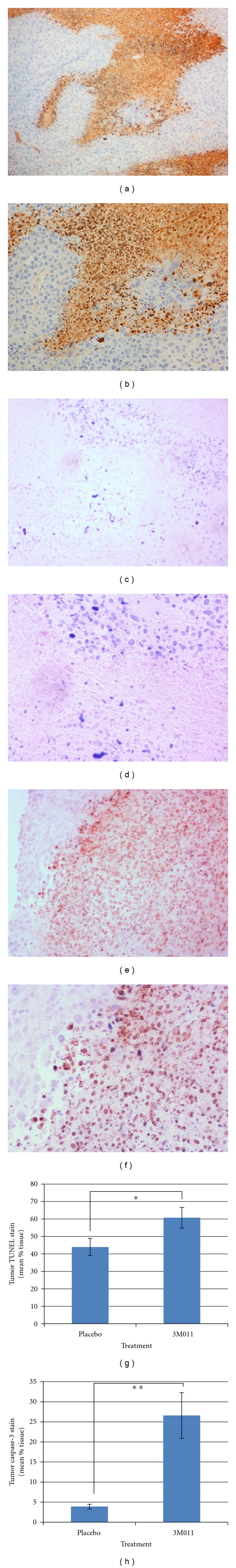
3M011 increases cell death in mouse RCC tumors. (a), (b) Representative example of a positive TUNEL patch of tissue at 100x (a) and 200x (b) magnification in a tumor treated 2 days prior with 3M011 imidazoquinoline; patchy tissue TUNEL positivity was also observed in placebo-treated tumors (not pictured). (c), (d) Representative example of negative cleaved caspase-3 immunostain at 200x (c) and 400x (d) magnification in a placebo-treated tumor, including a tissue patch with corresponding TUNEL positivity (bottom two-thirds of each picture). (e), (f) Representative example of diffusely positive cleaved caspase-3 immunostain at 200x (e) and 400x (f) magnification in a large tissue region corresponding to TUNEL positivity from a 3M011-treated tumor. (g), (h) Mean percentage of tissue staining positive for TUNEL (g) and cleaved caspase-3 (h) among tumors treated 2 days prior with placebo versus 3M011. *P < 0.05; **P < 0.001; error bars = standard error of the mean.

**Figure 5:**
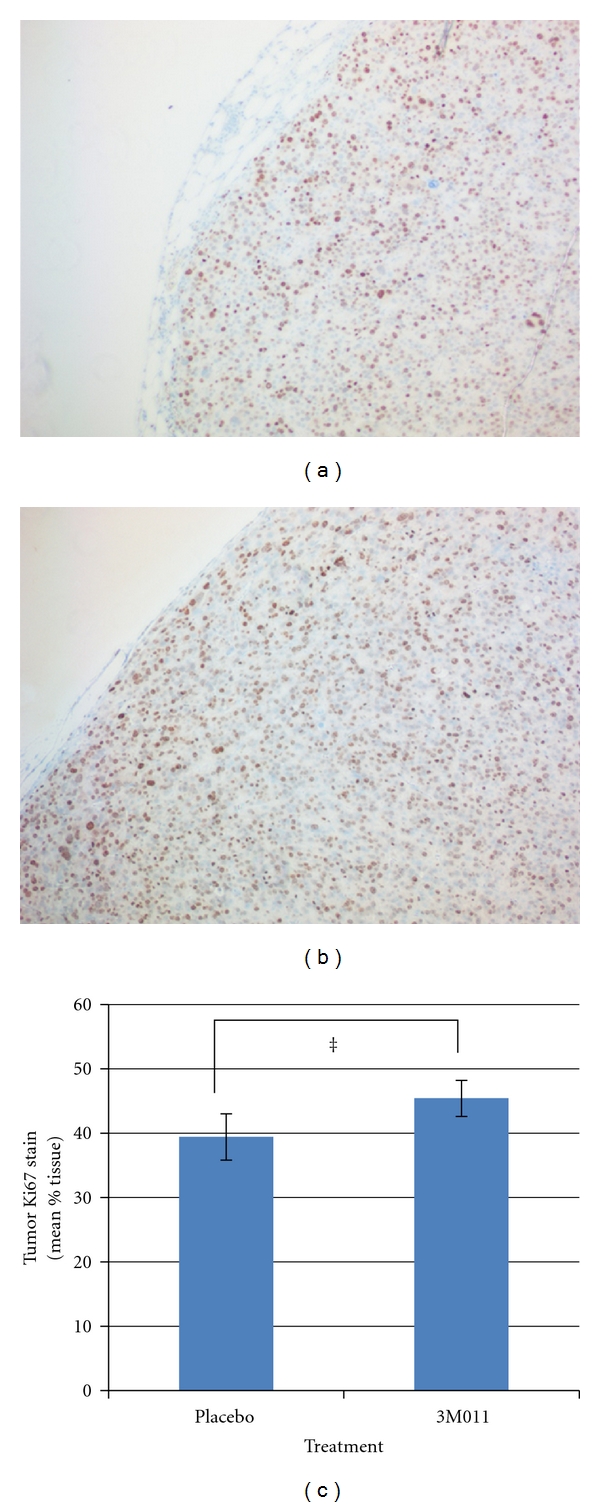
3M011 does not affect RCC tumor cellular proliferation. (a), (b) Immunohistochemical staining of RENCA RCC xenografts for Ki-67 cell proliferation marker following treatment 2 days prior with placebo (a) or 3M011 imidazoquinoline (b), representative examples at 100x magnification. (c) Mean percentage of tumor tissue with positive Ki67 staining 2 days after placebo versus 3M011 treatment. ^‡^ *P* = 0.21.

 TUNEL staining was used to assess the degree of cell death in treated mouse tumors as a nonspecific marker of necrosis and apoptosis. Mouse tumors had variable TUNEL positivity, ranging from 10% to 80% of the sectioned tissue areas, reflecting what appeared histologically as patchy regions of tumor necrosis (Figures 4(a) and 4(b)). However, TUNEL positivity was on average significantly more frequent in mice treated with imidazoquinoline compared to placebo (Figure 4(g)). Furthermore, only in imidazoquinoline-treated tumors did tissue regions corresponding to TUNEL positivity also demonstrate frequent positive staining for cleaved caspase-3 indicative of apoptosis (Figures 4(c)–4(f), and 4(h)). Specifically, the percentage of tumor tissue with positive cleaved caspase-3 staining was on average 26% in imidazoquinoline-treated tumors compared to just 4% in placebo-treated tumors (*P* < 0.001). No significant difference in cell proliferation based on Ki67 staining was observed in imidazoquinoline versus placebo-treated tumors (Figure 5).

### 3.3. Imidazoquinoline Effect on Intratumoral Leukocytic Infiltration

**Figure 6:**
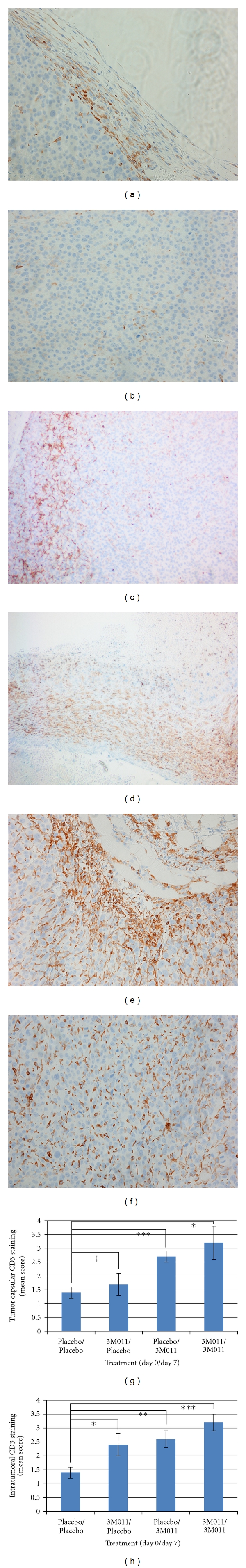
3M011 increases T-cell lymphocytic infiltration in RCC tumors. CD3 immunohistochemical staining of T-cell lymphocytes in RENCA RCC xenografts following treatment with placebo or 3M011 imidazoquinoline. (a) Representative RCC tumor from a placebo-treated mouse with mild T-cell lymphocyte collection at the tumor surface but little intratumoral penetration (200x magnification). (b) Center of tumor from (a) with infrequent T-cell lymphocytes (200x magnification). (c) Example of a placebo-treated tumor with moderate T-cell concentration at the tumor periphery but still little central tumor infiltration (100x magnification). (d), (e) Representative 3M011-treated tumors with large T-cell aggregates at the tumor edge (100x and 200x magnification, resp.); the bottom aspect of (d) represents necrotic tumor. Note the effective intratumoral penetration in (e). (f) Representative 3M011-treated tumor center showing diffuse infiltration of CD3-positive T-cell lymphocytes (200x magnification). (g), (h) Tumor capsular CD3 staining (i) and intratumoral CD3 staining (j) comparison in mice treated with placebo versus 3M011 on day 0 and day 7. *P < 0.05, **P < 0.01, ***P < 0.005, and ^†^ *P* = 0.52.

 To evaluate the effect of the imidazoquinoline treatment on leukocytic infiltration, mouse tumors were stained for immune cell protein markers CD3 (T-cell lymphocyte marker), B220 (B-cell lymphocyte marker), and CD-45 (pan-leukocyte marker) 2 days and 9 days after mouse treatment. In mice receiving placebo treatments only, tumors demonstrated mild-to-moderate (1-2+) T-cell lymphocytic infiltration by CD3 staining, mostly confined around the tumor edge with only sparse penetration into the central tumor (Figures 6(a)–6(c)). In contrast, with one exception, all mice receiving two imidazoquinoline treatments demonstrated large (3-4+) peritumoral aggregates of T-cell lymphocytes as well as diffuse (3-4+) infiltration into the central tumor (Figures 6(d)–6(h)). The one exception occurred in a tumor with only mild-to-moderate (1-2+) T-cell lymphocytic infiltration despite two imidazoquinoline treatments; notably, this one tumor was of large size and had minimal (<5% of tumor) apoptosis by cleaved caspase-3 staining, suggesting resistance to imidazoquinoline. Mice treated only once with imidazoquinoline also had increased T-cell lymphocytic infiltration relative to placebo-treated mice, but not to the extent as mice receiving two imidazoquinoline treatments (Figures 6(g) and 6(h)). Staining for the CD45 pan-leukocyte marker in all tumors correlated closely with CD3 staining (data not shown), supporting that T-cell lymphocytes constitute the primary bulk of RENCA tumor infiltrating leukocytes. B220 staining picked up only rare scattered single cells without differences in imidazoquinoline-treated and placebo-treated tumors (data not shown), supporting the relative lack of B-cell lymphocytic infiltration in RCC tumors and the imidazoquinoline-mediated immune response.

### 3.4. Imidazoquinoline Effect on Intratumoral Cytokine Production

**Figure 7:**
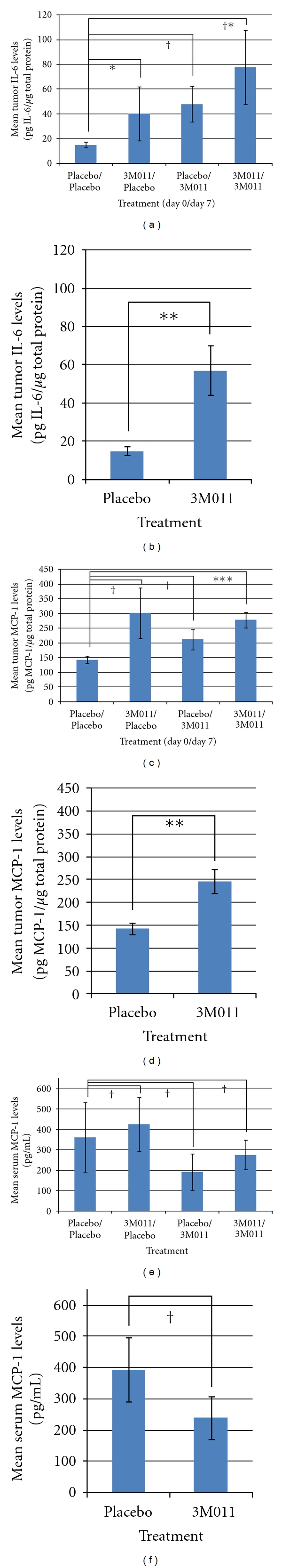
3M011 increases proinflammatory cytokine production in mouse RCC tumors but not in mouse serum. (a)–(d) Levels of intratumoral IL-6 (a), (b) and MCP-1 (c), (d) cytokines in mouse RCC xenografts on day 9 following treatment with placebo versus 3M011 imidazoquinoline on day 0 and day 7. (e), (f) Levels of serum MCP-1 in mice at the same time points. (b), (d), and (f) Compare mice receiving placebo on both day 0 and day 7 versus mice receiving 3M011 on one or both of these days. *P < 0.05, **P < 0.01, ***P < 0.005, and ^†∗^ *P* = 0.08, ^†^ *P* > 0.10. Error bars = standard error of the mean.

 Serum and tumor levels of proinflammatory cytokines, IL-6 and MCP-1, were assessed by ELISA 2 days and/or 9 days after treatment with imidazoquinoline. In tumor tissue, high levels of IL-6 (>3000 pg/100 mg tumor) were observed exclusively in imidazoquinoline-treated tumors, which on average had four times higher levels than placebo-treated tumors (*P* < 0.01) (Figures 7(a) and 7(b)). Similarly, levels of MCP-1 were on average nearly double in imidazoquinoline-treated tumors compared to controls (*P* < 0.01) (Figures 7(c) and 7(d)). Mice receiving two doses of imidazoquinoline had nearly double the IL-6 levels of single-dosed mice; however, this difference was not statistically significant (*P* = 0.26). Similarly, no significant differences in intratumoral MCP-1 levels were observed between once and twice dosing of imidazoquinoline (Figure 7(c)). In mouse serum, no differences in MCP-1 levels were observed following imidazoquinoline treatments compared to controls, (Figures 7(e) and 7(f)) and serum IL-6 remained undetectable below our ELISA assay limit of >30 pg/mL in all mice regardless of treatment.

## 4. Discussion

 Despite the existence of tumor-specific antigens recognizable by the host immune system, the ability of different cancers to evoke an immune response is quite variable, with most solid tumors being only weakly immunogenic [9, 10]. In contrast, RCC solid tumors are highly immunogenic with abundant T-cell lymphocytic infiltration, and immunotherapies such as IL-2 and IFN-alpha are among the most successful systemic treatments for RCC patients [7, 8]. However, only a minority of metastatic RCC patients respond to these treatments, and primary tumors are particularly resistant, suggestive of underlying RCC immunoevasion.

Dendritic cell inactivation may play a central role in cancer immunoevasion. Maturation of dendritic cells initiated by TLR7 activation is a well-known requisite for downstream activation of antigen-specific T-cell effector responses [13]. High levels of immature dendritic cells have been demonstrated in cancer patients and shown to cause T-cell unresponsiveness in animal tumor models [25–28]. In RCC primary tumors, immature dendritic cells may accumulate as a result of upregulated immunoevasive antigens on RCC cells, such as the B7 family of PD-1 receptor ligands (e.g., B7-H1) or immunosuppressive cytokines such as IL-10 [29, 30]. Therapeutic targeting of dendritic cell activation in RCC patients may thus provide a novel strategy for countering immunoevasion in both primary tumors and systemically disseminated disease.

Imidazoquinolines are synthetic TLR7/TLR8 agonists and potent dendritic cell activators with established anticancer activity in both patients and mice [23, 31]. The prototypical agent, imiquimod, has had clinical success in the topical treatment of dermatologic malignancies [20–22]. In addition, we have previously described *in vivo* efficacy of the related imidazoquinoline, 3M011, in suppressing bladder cancer tumorigenicity and RCC pulmonary colonization in a metastatic mouse model [23, 24]. In our current study, we have employed a syngeneic primary tumor xenograft mouse model to show that 3M011 also has *in vivo* suppressive activity against established RCC tumors. Specifically, 3M011 intratumoral injection decreased mouse RCC tumor size by approximately 50% relative to placebo controls. This growth inhibition was apparent after two days following a single dose, and repeat dosing one week later compounded the effect. Growth inhibition was independent of pretreatment tumor size, as initial treatment of even large tumors (~5% of the mouse total body mass) still resulted in partial tumor regression. The durability of tumor responses with repeat dosing observed in this study is in contrast to the acquired TLR7 agonist resistance described by Bourquin et al. [16]. These authors demonstrated that a single injection of imidazoquinoline induced tolerance to repeat dosing, associated with suppression of proinflammatory cytokine production. TLR7 tolerance lasted up to 5 days in their study, suggesting a wider dosing interval as used in this study may be necessary.

 With regards to drug toxicity, intratumoral injection of imidazoquinoline was well tolerated by mice in this study and appears to have therapeutic efficacy against RCC below doses needed for systemic toxicity. The dose used here (2.5 mg/kg) is lower than doses commonly studied for imidazoquinolines in other cancer models (≥5 mg/kg) and subcutaneous doses reported to cause systemic toxicity (≥10 mg/kg) [32]. The most commonly reported systemic side effect of TLR7 agonists is anorexia [33], and imidazoquinoline-treated mice in this study maintained their weights well. TLR7 agonists in mice have additionally been documented to cause “cytokine syndrome,” manifested as shivering, and raised fur due to elevated systemic cytokines [32]. This syndrome was not observed in our study, and no increase in serum proinflammatory cytokine levels could be detected after imidazoquinoline treatment despite the increased intratumoral levels.

 The molecular and cellular events responsible for imidazoquinoline-mediated tumor suppression, including the role of the immune system, are not well characterized. While the ability of imidazoquinolines to directly induce tumor cell apoptosis likely plays a role, it is generally believed that the clinical anticancer activity is mediated primarily by activation of a robust anticancer immune response resulting from potent dendritic cell activation [34]. In the current study, we found a significant increase in cell death via apoptosis in imidazoquinoline-treated tumors relative to controls, but without change in viable tumor cell proliferation, supporting induction of apoptosis as the primary mechanism of RCC tumor growth inhibition. This finding is consistent with prior reports from our group, and others showing imidazoquinolines may induce apoptosis in multiple cancer types, including in the absence of immune cells [23, 31]. In addition, we found that imidazoquinoline treatment dramatically enhances T-cell lymphocyte infiltration along the periphery and within the central bulk of RCC tumors, as well as intratumoral production of proinflammatory cytokines, consistent with upstream dendritic cell activation. Whether this immune response contributes to the tumor growth suppression observed in this study requires further study. While we observed rapid timing of tumor suppression consistent with an innate immune response, imidazoquinolines may additionally exert antitumor activity by adaptive immune system activation. For example, Redondo and colleagues have shown in a melanoma mouse model that imidazoquinoline pretreatment combined with tumor cryoablation to enhance antigen presentation can prevent tumorigenesis of cancer cells reintroduced into mice weeks later, supporting a role for memory T cells [35].

Lack of tumor growth suppression with repeated 3M011 treatments was observed in a single mouse in this study. In clinical neoplasias, imidazoquinoline therapeutic response has been associated with increased CD8+ T-cell lymphocyte tumor infiltration [36]. Consistent with this observation, the nonresponsive mouse in our study demonstrated less T-cell tumor infiltration than responsive mice. Clinical resistance to imidazoquinoline has also been correlated with increased intratumoral levels of immunosuppressive T_reg_ cells characterized by the CD4+/FOXP3+/CD25+ phenotype which are believed to promote immunoevasion by inhibiting cytotoxic T-cell responses [37–39]. Recent investigation in mouse models has shown that imidazoquinoline therapy increases the number and activity of T_reg_ cells in part through T_reg_ expression of TLR7; however, opposite findings are reported in cervical cancer patients [40–42]. Imidazoquinoline responsiveness may thus depend on a complex balance between opposing activities of cytotoxic T cells and immunosuppressive T_reg_ cells. Subtype characterization of infiltrating T-cell lymphocytes in imidazoquinoline-treated RCC tumors will be useful to elucidate the role of the immune system and perhaps enable more accurate prediction of therapeutic response.

 In conclusion, this study demonstrates imidazoquinoline TLR7 agonist to be an effective therapy against established murine RCC primary tumors. Similar to its well-characterized effects in dermatologic malignancies, imidazoquinoline treatment enhances RCC intratumoral T-cell lymphocytic infiltration and proinflammatory cytokine production while inhibiting RCC tumor growth via induction of cancer cell apoptosis. These effects may relate to the ability of imidazoquinolines to stimulate potent dendritic cell activation via TLR7 stimulation leading to a a downstream cytotoxic T-cell lymphocyte immune response. These findings corroborate our previous demonstration of biologic efficacy against disseminated RCC cells and warrant further investigation to determine whether TLR7 activation may have clinical utility in therapeutically targeting both the primary tumor and systemic disease in RCC patients.
